# Integrin and FAK Regulation of Human Pluripotent Stem Cells

**DOI:** 10.1007/s40778-017-0100-x

**Published:** 2017-10-13

**Authors:** Loriana Vitillo, Susan J. Kimber

**Affiliations:** Division of Cell Matrix Biology and Regenerative Medicine, Faculty of Biology Medicine and Health, University of Manchester, Michael Smith Building, Oxford Rd, Manchester, M13 9PT UK

**Keywords:** Pluripotent stem cells, Human embryonic stem cells, Stem cell niche, FAK, Integrin signaling, Pluripotency networks

## Abstract

**Purpose of review:**

Human pluripotent stem cells (hPSCs) are anchorage-dependent cells that can be cultured on a variety of matrices and express integrins and the machinery for integrin signaling. Until recently, there has been limited understanding of exactly how integrin signaling regulates pluripotent stem cell (PSC) behavior. This review summarizes our knowledge of how integrins and focal adhesion kinase (FAK) regulate different aspects of hPSC biology.

**Recent findings:**

The latest research suggests that mouse and human embryonic stem cells utilize similar integrin signaling players but with different biological outcomes, reflecting the known developmental difference in their pluripotent status. Notably, attachment cues via FAK signaling are crucial for hPSCs survival and pluripotency maintenance. FAK may be found cortically but also in the nucleus of hPSCs intersecting core pluripotency networks.

**Summary:**

Integrins and FAK have been consigned to the conventional role of cell adhesion receptor systems in PSCs. This review highlights data indicating that they are firmly integrated in pluripotency circuits, with implications for both research PSC culture and scale up and use in clinical applications.

## Introduction

Human embryonic stem cells (hESCs) derived from cells of the inner cell mass of the blastocyst and induced pluripotent stem cells (iPSCs) reprogrammed from somatic cells to an ESC-like state have attracted much interest in recent years because of their promise in understanding early human development, for modeling monogenic diseases, and for producing cell-based therapies. In order to utilize these pluripotent stem cells (PSCs) to their full potential, we need to be able to culture them stably with minimal death or differentiation, retaining their plasticity and responsiveness together with the ability to undergo repeated cell division (self-renewal). Thus, the development of appropriate culture conditions (including those suitable for clinical use) has generated a thriving industry.

Although the importance of cell-cell interactions for stable hESC growth is well established, the role of cell-substrate interactions is not fully understood. There is documented evidence of a role for integrins [1, 2], and knowledge of the integrin complement has often been exploited, matching them to more favorable single extracellular matrix (ECM) substrates for culture [2–5]. However, the machinery and signaling pathways linking these interactions to the pluripotent state are still not entirely clear. In this review, we summarize findings relating to the stable substrate growth of PSCs and the role of integrins and focal adhesion kinase (FAK) in the transduction of signals from the substrate.

## Into the Pluripotent Stem Cell Niche

In vivo, adult stem cells reside as quiescent cells in specific locations of many human organs referred to as “niches” [6] which offer a protected and finely controlled environment for their long-term maintenance [7]. Although the pluripotent stem cell niche in vivo is only a transient developmental stage [8], in vitro, pluripotent stem cells can grow indefinitely under controlled culture conditions, and it has been found that hESCs may also produce their own niche of surrounding feeder cells [9, 10] and ECM [4].

A specialized niche for ESCs requires a set of extracellular cues, both chemical and physical, that sustains the core-circuitry linked to proliferation, survival, and pluripotency. While the roles of soluble factors such as FGF and TGFβ family growth factors and, to a certain extent, cell-cell interaction have been extensively studied in controlling stem cell behavior, the unique contribution of ECM-dependent pathways has been less investigated.

In the context of the stem cell niche, the ECM plays three main roles: (i) cell anchorage, (ii) growth factor and morphogen reserve, and (iii) a mediator of biomechanical stiffness including through modification of its density and elasticity [11]. In a regular stem cell niche, these three roles are all present and linked making the ECM regulation of stem cells of considerable importance. ECM components have been found to bind growth factors and morphogens with high affinity, thus creating local gradients of concentrated factors adjacent to their cell surface receptors [12]. Other ECM domains bind to predominantly integrin receptors. By these means, the ECM acts as a scaffold that simultaneously and spatially concentrates integrins, growth factors, and growth factor receptors, thus activating common downstream pathways known to crosstalk and activate one another [12].

Over the years, several groups have developed methods that recreate an artificial pluripotent stem cell niche. A turning point was achieved with the direct culturing of hESCs on purified ECM molecules, such as vitronectin, fibronectin, and laminin known to be secreted by both MEFs and hESCs [2–4, 13–18].

## Integrin Receptors in ES Cells

Integrins are heterodimers formed by combinations of 18α subunits and 8β subunits, giving a total of 24 glycoproteins with distinct binding preferences, functions, and knockout phenotypes [19]. Structurally, all integrins are composed of a large extracellular domain, a single transmembrane domain, and a short cytoplasmic tail [20]. The cohort of integrins expressed by a cell determines the type of ECM-cell interaction, which can dramatically change cell behavior.

## Murine ESC Integrins

β1-integrin has been associated particularly with stemness in adult and embryonic stem cells [21]. It is present throughout preimplantation embryonic development [22], and β1 integrin-null mice show survival, proliferation, and migration defects of early embryonic tissues developing from the inner cell mass [23] and die after implantation [24]. However, ECM components used for culturing undifferentiated mESCs (e.g., gelatin, or poly-D-lysine) do not all activate integrins, and it has been argued that electrostatic interaction is more important than integrin-matrix binding [25], but it is clear that integrin-substrate interactions play a role. Indeed, by using 3D hydrogels carrying integrin selective peptides (e.g., RGD), others have shown a direct correlation between activation of 4 mESC integrins (α5β1, αvβ1, α6β1, and α9β1) and an increase in pluripotency markers [26]. Integrin α5β1 is considered the fibronectin receptor, binding fibronectin via the RGD peptide [27]. Hayashi et al. [25] showed that growing mESCs on integrin-activating substrates, such as fibronectin or vitronectin, negatively correlated with both their proliferation and self-renewal. This is supported by the finding that integrin activation in mESCs increases ERK [25], known to inhibit the self-renewal of these cells [28]. Moreover, others showed that only laminin-511, but not other laminin isoforms, supports mESCs self-renewal, via mainly α6- and αVβ1integrin engagement [29]. These combined data indicate that mESCs do not require activation of integrin signaling for their self-renewal. On the contrary, integrin activation generally induces differentiation of mESCs [25] and negatively regulates pluripotency (with the apparent exception of α6- and αVβ1integrin on laminin511), which is sustained predominantly by soluble factors and through attachments to adjacent cells.

## Human ESC Integrins

Under current culture regimes, hESCs are maintained in an undifferentiated state in the absence of feeder cells by the use of single ECM substrates. Because many ECM molecules activate a particular subset of integrins, even on single substrates several integrins could be engaged, although certain heterodimers will dominate.

Although hESCs tend to express a series of integrins [2, 4, 5], a restricted number has been found to be functional [30]. Indeed, hESCs on Matrigel (a laminin-rich ECM mixture) have been reported to adhere by αVβ3, α6β1, and α2β1, assessed by interfering with direct attachment using blocking antibodies and peptides [30]. However, single ECM substrates rather than mixed substrates are preferable to assess integrin function. HESCs bind to fibronectin selectively via its established preferential integrin receptor, α5β1 [2, 3] while on vitronectin, αVβ5 is activated [2]. HiPSCs similarly express integrin subunits, particularly α5, α6, αv, β1, and β5, and attach and proliferate on Matrigel via β1 integrins and on vitronectin using αVβ5 and β1 integrins [31]. Finally, again β1 integrin, in combination with α6, mediates the strong binding of hESCs and hiPSCS to recombinant laminin-511 [2, 18].

Overall, the repertoire of integrins expressed by hESCs and hiPSCs on selective ECM components has been shown to be restricted and substrate-biased.

## Integrin Signaling: a Glance at the Focal Adhesion Kinase Pathway

In many cells, small focal complexes at the leading edge of the cells mediate the first attachment to the ECM. Subsequently, these complexes stabilize, generating focal adhesions (FA) in parallel to the retraction of the lamellipodium. FA provide strong binding to the ECM and the cytoplasmic recruitment of a whole network of interacting proteins [32•] including actin fibrils [33].

Following the binding of talin and kindlin to the β-integrin tail (and its activation by ligand binding), the first ECM-cytoskeleton link is established through these structural connecting proteins [34]. Because activation promotes the clustering of the integrins, multiple talin-mediated connections to actin fibers create a robust ECM-cytoskeleton interaction and provide a starting point for the assembly of the adhesome.

One of the first signaling proteins recruited at the adhesome is focal adhesion kinase (FAK), a non-receptor tyrosine kinase that binds talin and paxillin via its C-terminal focal adhesion-targeting (FAT) domain [35]. FAK is a crucial FA protein that intersects many pathways and triggers the cellular response to ECM by acting as a signaling integrator. It contains three main domains and two linkers. The C-terminal FAT domain [36] is responsible for localization of FAK to focal contacts and contains hydrophobic binding sites for paxillin. One of the tyrosine residues of FAK that is in the FAT domain, Tyr 925, when phosphorylated stabilizes a conformation that allows binding of the SH2 domain of Grb2 [37]. The tyrosine kinase domain is bilobed and contains an ATP binding site responsible for kinase activity. In order to be fully activated, Tyr576 and Tyr577 within the activation loop need to be phosphorylated by Src [38]. The N-terminal band 4.1 Ezrin Radixin Moesin (FERM) domain is trilobed as for many other FERM domains [39] and responsible for nuclear localization, auto inhibition of FAK kinase activity, and facilitation of survival pathways [36]. The linker region between the FERM and kinase domain contains the autophosphorylation site in Tyr397 (Y397). Autophosphorylation at Y397 is the first event that activates FAK after recruitment to focal contacts, creating an SH2-binding site for Src and PI3K [40]. It also contains proline-rich “PxxP” motifs that are recognized by SH3-binding proteins like Src [41]. The linker region between the kinase and FAT domains also contains two SH3-binding sites for scaffolding proteins like p130Cas [42].

Following integrin activation, FAK is autophosphorylated at Y397 permitting a low level of kinase activity and creating an SH2 binding site for proteins like the p85 regulatory subunit of PI3K [43] and Src-family protein tyrosine kinases, mainly Src itself [37, 40]. The FAK/Src complex allows phosphorylation of the activating loop of the kinase domain by Src, leading to full FAK activity [37]. Active FAK in turn phosphorylates Src, which propagates the signal and promotes the aggregation of adhesome proteins around FAK [44]. For example, after phosphorylation on Tyr 925 by Src, the Grb2-SOS complex binds FAK, generating crosstalk between ECM and growth factor signals [40]. Grb2 transduces the signal to RAS/Raf/MEK and finally ERK, while Src phosphorylates p130Cas, associated with FAK, and upstream of JNK: both cascades converge into cell cycle progression [40]. Notably, FAK activation downstream of α5β1-integrin can co-operate with signals from the FGF-2 receptor for the activation of PI3K and subsequently activation of both the Akt and the RAS/MAPK cascade [45]. FAK also plays a pivotal role in the spreading, protrusion, and invasion of many but not all cells [46–48]. Overall, integrin activation of the FAK-Src complex induces signaling cascades connected with survival, motility, and cell cycle progression.

## Life and Death by FAK

One of the pivotal roles of FAK in the cell is the control of survival. In epithelial cells, loss of attachment to the ECM triggers a special form of apoptosis, named anoikis [49]. Anoikis is executed by the mitochondrion downstream of an integrin signaling cascade [50]. Indeed, it is rescued if the cells are re-plated on fibronectin or on culture dishes coated with β1-integrin antibodies [51]. Moreover, constitutive expression of FAK protects the cells from anoikis [52], while blocking the interaction of FAK with the cytoplasmic tail of β1-integrin leads to high levels of anoikis [53].

The cellular control of survival is principally mediated by PI3K [54, 55•], a survival pathway confirmed in mESC [56, 57], hESCs [58, 59], and hiPSCs [60]. In mESCs, PI3K acts to inhibit both MAPK/ERK required for differentiation and GSK3β, the latter leading to upregulation of Tbx3 and Nanog [55•, 61, 62]. Blocking FAK or inhibiting Src reduces the activation of the p85 subunit of PI3K as well as phosphorylation of Akt at Ser473, and promotes apoptosis of fibroblasts. Enforced activation of β1-integrin or overexpression of PI3K p110 catalytic subunit rescues cells from apoptosis caused by dominant negative FAK, thus showing that FAK acts upstream of PI3K/Akt in the suppression of apoptosis by β1-integrin [63]. The tail region of integrins β1 and β3 can bind the linked integrin kinase (ILK), which in turn appears to activate Akt [64]. Overexpression of ILK can also prevent anoikis [65] in some settings. However, ILK does not have classic catalytic residues and it is considered to be a pseudokinase; therefore any role in regulating Akt, or in formation of cell-ECM contacts, is likely due to ILK interaction with molecular partners Pinch and Parvin (IPP complex) [66].

So, what pathways lie downstream of Akt to prevent anoikis? Activated Akt induces the transcription factor NFkB, which up regulates pro-survival Bcl-2 proteins (Bcl-2 and Bclxl) [67]. Overexpression of Bcl2 in cells without integrin α5β1 blocks anoikis [68]. Interestingly, the translocation of NFkB to the nucleus, also mediated by ERK (in the MAPK cascade), seems to require integrin engagement [69].

Another target of Akt is MDM2, involved in the proteasome-mediated degradation of p53. The p53 protein is an effector of anoikis, and this process is regulated by FAK: in its absence, there is an increased p53-dependent apoptosis [70]. Moreover, Ilic et al. showed that mutation in the DNA-binding region of p53 did not interfere with the induction of apoptosis, instead p53 protein acts directly on the caspase cascade in its execution phase [70].

Investigation of the FERM domain of FAK has revealed new roles for FAK in the nucleus. The FERM domain correlates with nuclear localization of FAK [71], and Goluboskaya et al. [72, 73] showed that it interacts directly with the N-terminal transactivation domain of p53 to inhibit its activity. Under reduced cell adhesion, FAK leaves FAs and localizes in the nucleus to act in a kinase-independent manner facilitating survival [74]. In particular, FAK’s FERM domain functions as a scaffold for MDM2-dependent p53 ubiquitination, thus increasing p53 turnover as a survival strategy [74].

Overall, although the general characteristics of FAK survival-signaling involve similar molecules in different cells, it is more likely that preferred pathways are activated in a cell type-specific manner.

## Integrin Signaling in ES Cells

Currently, detailed reports of integrin/FAK signaling mechanisms operating in ESCs are scarce compared to the literature on adult cell types. However, several studies have mapped the activity of some key downstream signaling players following integrin engagement in ESCs, paving the way to our understanding of how ESC integrin signaling responds to ECM cues in the context of a pluripotent niche.

Integrin-activating substrates, such as vitronectin, laminin, and fibronectin, are effective for maintaining self-renewal of undifferentiated hESCs under fully defined conditions [2, 3, 18] as described above. By contrast, integrin engagement and integrin signaling activation in mESCs induce the opposite effect and trigger differentiation [25, 75•]. Indeed, culturing mESCs on fibronectin or laminin precipitates differentiation via activation of FAK and Akt, while collagen support pluripotency [25]. In line with these findings, it was shown that α6β1 and α3β1 integrin engagement with laminin facilitates mESC differentiation towards an epithelial lineage [75•]. Activated α6-integrin interacts with the adaptor protein CD151 inducing α6β1 internalization, in turn eliciting a FAK/Akt cascade that leads to phosphorylation of Erk and ultimately cell fate specification [75•]. Furthermore, the role of integrin signaling in mESC fate has been addressed by adding ECM ligands to magnetic beads [76]. MESCs incubated with RGD, fibronectin, or laminin-coated beads decreased Oct-4 expression while beads coated with the cell-cell adhesion receptor E-cadherin did not decrease Oct-4 [76]. Thus, pluripotency in mESC and hESC seems similarly affected by cell-cell contact cues but not integrin engagement, although the bead application does not provide the same vectorial substrate presentation as a culture surface. In addition to stimulating differentiation, integrin engagement in mESCs plays a role in cell cycle regulation [77]. Addition of fibronectin to the medium of mESCs cultured on gelatin induced a FAK/Src/Calvelolin/RhoA/PI3K/Erk1/2 pathway that supports proliferation [77].

In hESCs, however, even under different culture conditions, activation of similar integrin signaling components does not halt but rather supports pluripotency, self-renewal, and survival [5, 78, 79, 80••]. The evidence available suggests that mESCs differ from hESCs not just in their response to soluble morphogens, like LIF and BMPs, but also in the outcome of the integrin signaling responses downstream of attachment to the ECM (Table 1).

**Table 1:** Current knowledge of integrin signaling in mouse versus human ESCs

Features of integrin signaling	mESc^a^	hESc^a^
Present in focal adhesions	✕	✕
Integrin-activating substrates maintain pluripotency	✕(✓)	✓
Integrin signals transduced through FAK	✓	✓
Integrins transduced through PI3K/ Akt	✓	✓
Integrins transduced through ILK	✓	✓
Integrins transduced through ERK	✓	✓
Integrin engagement supports proliferation	✓	✓
Integrin/FAK activation inhibit anoikis	N/a	✓
Integrin/FAK activation inhibit hypercontractility	N/a	✓
Nuclear FAK	N/a	✓

✓ yes, ✕ no, *N/a* not available

^a^Referring to self-renewing undifferentiated culture conditions

There is a general agreement that adherence to ECM substrates that engage integrins leads to activation of both FAK and AKT in hESCs [5, 78, 79, 80••]. Not surprisingly, the modulation of the integrin signaling seems to vary depending on the ECM and culture conditions employed in these studies. For example, FAK, AKT, and ERK were more strongly activated in hESCs grown on recombinant E8 fragments of laminin than on vitronectin or fibronectin, which however all supported their phosphorylation [78, 79, 80••]. However, one report concluded that integrin/FAK signaling is only activated during differentiation of hESCs, while nuclear FAK maintains the pluripotency circuits [81••]. An explanation for this discrepancy can be found in the dynamic of FA formation. Focal adhesions are not present in pluripotent stem cells [3, 80••]—rather, integrin and associated molecules are found in puncta around the cell surface. Antibody activation of β1-integrin caused FAK to be prominently at the membrane, suggestive of co-localization with focal adhesions, while pluripotency-associated marker OCT4 decreased [81••]. Thus, hESCs already express β1-integrins and key focal adhesion components, such as paxillin, but these only assemble into focal adhesions upon differentiation [80••]. Therefore, integrin/FAK signaling seems non-canonical in hESCs, only to switch into its typical “adult” signaling following loss of pluripotency.

Such singular and dynamic integrin signaling in hESCs is highlighted by studying the behavior of integrin/FAK signaling in cells grown under both self-renewing and differentiating conditions.

## Multiple Roles for FAK in Human Pluripotent Stem Cells

Although we are only starting to dissect FAK’s regulation of hESC behavior, initial evidence points to important and varied roles in the context of pluripotent stem cells.

Afrikanova et al. [82] have looked at the role of FAK during derivation of β–cells from hESCs. They showed that inhibition of FAK or the FAK/Src complex improved endocrine specification, as well as inhibiting progenitor proliferation, by suppressing Smad2/3 [82]. These findings, in the context of differentiation-inducing culture conditions, demonstrate that FAK supports Smad2/3 signaling, pivotal in regulating the balance between pluripotency and differentiation [59]. This may result from FAK signaling crosstalk with the TGF-β cascade [82]. That integrin signaling poses a block towards differentiation of hESCs was also indicated by a report showing that inhibition of ILK, but not FAK, during endoderm differentiation reduced FAK and AKT phosphorylation while increasing SOX17-positive cells [79]. It is worth noting that FAK Y397, upstream of the PI3K/AKT cascade, was dephosphorylated after ILK inhibition, making it difficult to exclude its involvement in the activation of AKT. Moreover, these experiments were performed on cells grown on Matrigel, so activation of receptors for a number of ligands may affect downstream FAK/ILK/AKT transduction.

It is important to keep in mind that hESCs subject to varied differentiation protocols will interact with the ECM in a multitude of ways given their dynamic transitioning cell state and according to the specific cocktail of extrinsic factors. Indeed, research has focused on how to better exploit integrin signaling during diverse differentiation protocols, particularly relevant for clinical applications [83].

However, investigating FAK signaling in undifferentiated hESCs grown on a single ECM component, fibronectin, confirmed that FAK transduces integrin engagement through AKT to keep the balance of hESC phenotype towards self-renewal [80••]. Indeed, inhibition of either integrins or FAK is enough to suppress AKT and increase p53, ultimately driving the cells out of the undifferentiated state, as demonstrated by downregulation of *OCT4* and *NANOG* and increase in differentiation markers [80••]. The fact that the inhibition of FAK alone induced the exit from pluripotency in cells kept in self-renewing media suggests how unique and profound the contribution of ECM cues to the maintenance of hESCs is.

**Fig. 1:**
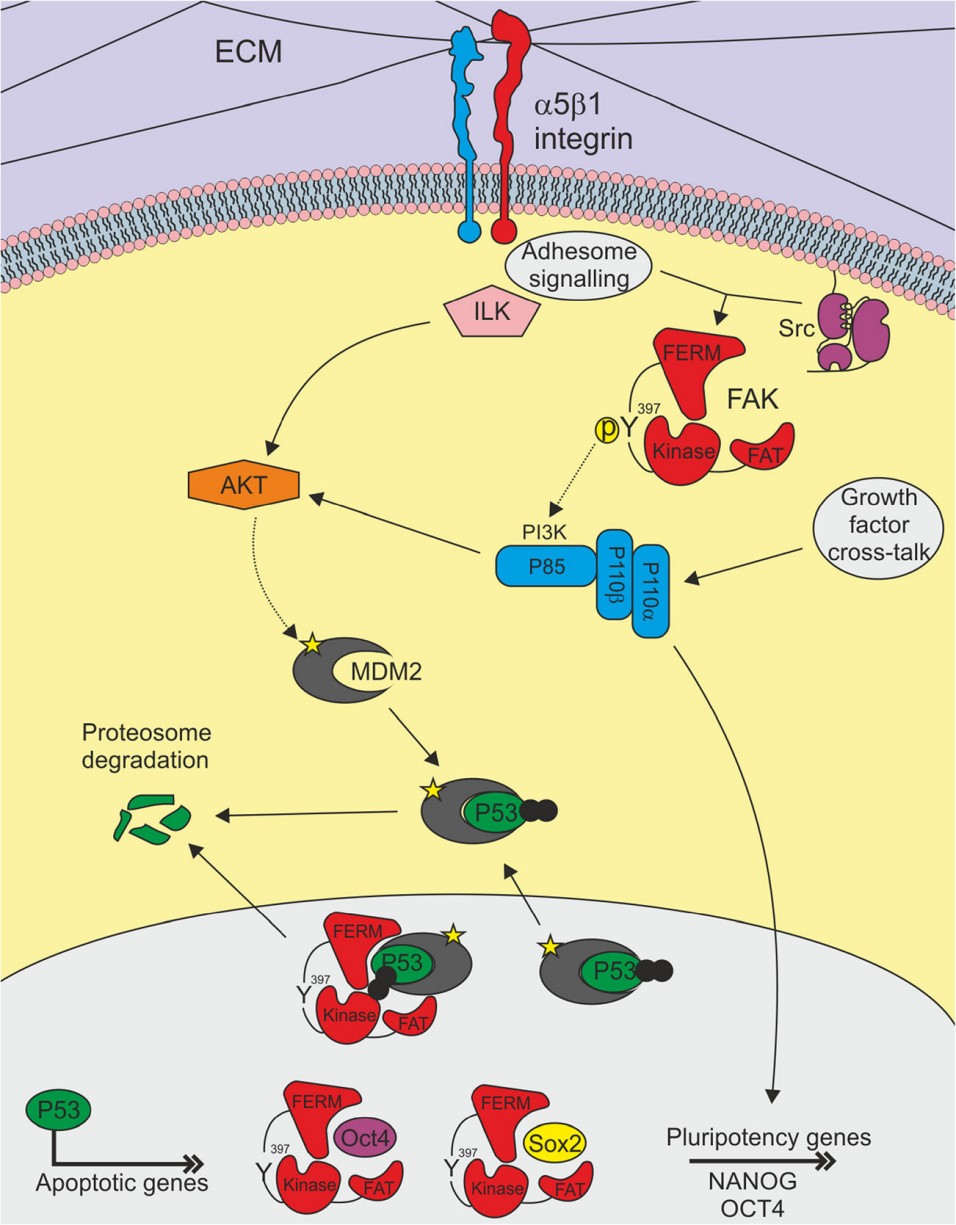
Diagram illustrating FAK signaling in hESCs. Stable ECM/integrin interaction induces activation of FAK, phosphorylated at Y397, at the cell surface, initiating the transduction of attachment cues to the cell. In turn, FAK Y397 activates the PI3K survival cascade, leading to phosphorylation of AKT and its downstream target MDM2. This allows MDM2 to continuously ubiquitinate p53 targeting it for proteosome degradation and maintaining low levels so that it cannot induce differentiation or apoptosis. In addition, FAK is localized in the nucleus where it binds pluripotency factors but can also exert its scaffolding roles in support of MDM2

However, a key function of FAK in hESCs is consistent with its well-known survival role in the cell: FAK transduces integrin engagement to a PI3K/AKT/MDM2/p53 cascade that suppresses a caspase-dependent anoikis (80••; Fig. 1). In addition, FAK supports adhesion to the ECM and suppresses hypercontractility [80••], which is in accord with FAK’s known role in mechanotransduction [46, 48].

As we have seen, FAK not only works as a kinase in the adhesome, it also acts as a scaffold in the nucleus [74]. Interestingly, in undifferentiated hESCs, where focal adhesions are not formed but FAK signaling is active [80••], FAK is found at the membrane, in the cytosol, and dephosphorylated FAK is also relatively high in the nucleus [80••, 81••]. Moreover, after kinase inhibition, the total pool of FAK decreased [80••], while in adult cell types, where FAK is not normally nuclear, it accumulates in the nucleus to scaffold the turnover of p53 by MDM2 and supports survival under stress [74]. These data suggest that, in hESCs, nuclear FAK likely already supports turnover of p53 in standard conditions, but when detachment or loss of integrin signaling is sensed, it quickly decreases, allowing sustained p53 activity and progress to anoikis or differentiation [80••].

Overall, these findings suggest that hESCs utilize integrin/FAK signaling in non-canonical ways that we are just starting to uncover. A fascinating example of this comes from the fact that a nuclear pool of FAK in hESCs might play other roles connected not with survival, but with pluripotency, similar to findings in cancer. Cancer cells share many characteristics with ESCs, such as the expression of the self-renewal factor Nanog [84]. In relation to FAK, it was shown that Nanog binds to the FAK promoter and FAK binds and phosphorylates Nanog in the nucleus [84]. Not only is FAK in the nucleus of stable hESCs [80••, 81••] but it was shown to co-immunoprecipitate with SOX2 and OCT4 but not NANOG [81••], and our unpublished pull-down experiments confirm this finding. This suggests that the intersection of FAK signaling with the pluripotency circuits may be more complex than previously assumed (Fig. 1).

Evidence to date indicates that the ECM significantly controls hESC life, death, and fate via integrin/FAK signaling for survival, adhesion, and supporting pluripotency signaling networks by means of canonical and non-canonical mechanisms that are worth further investigation.

## Conclusions

Both mESCs and hESCs rely on FAK and integrin signaling as part of the repertoire of signals guiding and expediting their differentiation. FAK has multiple roles in ESCs, such as in survival, proliferation, anchorage, contractility, and support or inhibition of self-renewal. The available literature points to different roles for integrins and FAK between hESCs and mESCs, possibly because the former are at a primed state ready to differentiate on induction and this may be a switch point triggering hESC dependence on FAK for maintenance as healthy pluripotent and dividing stem cells. Moreover, it is possible that integrin/FAK interaction with the cytoskeleton internally and via crosstalk with growth factor and other exogenous signaling pathways contribute to a cell state receptive to following different lineage pathways as instructed by other dominant factors. The evidence of FAK in the nucleus of hESCs is fascinating and would need further work to determine if similar pathways are operating to those shown in cancer cells.

## References

[1] Ohgushi M, Matsumura M, Eiraku M, Murakami K, Aramaki T, Nishiyama A (2010). Molecular pathway and cell state responsible for dissociation-induced apoptosis in human pluripotent stem cells. Cell Stem Cell.

[2] Braam SR, Zeinstra L, Litjens S, Ward-van Oostwaard D, van den Brink S, van Laake L (2008). Recombinant vitronectin is a functionally defined substrate that supports human embryonic stem cell self-renewal via alphavbeta5 integrin. Stem Cells.

[3] Baxter MA, Camarasa MV, Bates N, Small F, Murray P, Edgar D (2009). Analysis of the distinct functions of growth factors and tissue culture substrates necessary for the long-term self-renewal of human embryonic stem cell lines. Stem Cell Res.

[4] Soteriou D, Iskender B, Byron A, Humphries JD, Borg-Bartolo S, Haddock MC, et al. Comparative proteomic analysis of supportive and unsupportive extracellular matrix substrates for human embryonic stem cell maintenance. J Biol Chem. 2013;288(26):18716–31. 10.1074/jbc.M113.463372 PMC3696646 23658023

[5] Rodin S, Antonsson L, Niaudet C, Simonson OE, Salmela E, Hansson EM (2014). Clonal culturing of human embryonic stem cells on laminin-521/E-cadherin matrix in defined and xeno-free environment. Nat Commun.

[6] Nishikawa SI, Osawa M, Yonetani S, Torikai-Nishikawa S, Freter R (2008). Niche required for inducing quiescent stem cells. Cold Spring Harb Symp Quant Biol.

[7] Scadden DT (2006). The stem-cell niche as an entity of action. Nature.

[8] Lensch MW, Daheron L, Schlaeger TM (2006). Pluripotent stem cells and their niches. Stem Cell Rev.

[9] Bendall SC, Stewart MH, Menendez P, George D, Vijayaragavan K, Werbowetski-Ogilvie T (2007). IGF and FGF cooperatively establish the regulatory stem cell niche of pluripotent human cells in vitro. Nature.

[10] Moogk D, Stewart M, Gamble D, Bhatia M, Jervis E (2010). Human ESC colony formation is dependent on interplay between self-renewing hESCs and unique precursors responsible for niche generation. Cytometry A.

[11] Brizzi MF, Tarone G, Defilippi P (2012). Extracellular matrix, integrins, and growth factors as tailors of the stem cell niche. Curr Opin Cell Biol.

[12] Harada M, Murakami H, Okawa A, Okimoto N, Hiraoka S, Nakahara T (2009). FGF9 monomer-dimer equilibrium regulates extracellular matrix affinity and tissue diffusion. Nat Genet.

[13] Ilic D (2006). Culture of human embryonic stem cells and the extracellular matrix microenvironment. Regen Med.

[14] Fu X, Xu Y (2011). Self-renewal and scalability of human embryonic stem cells for human therapy. Regen Med.

[15] Miyazaki T, Futaki S, Hasegawa K, Kawasaki M, Sanzen N, Hayashi M (2008). Recombinant human laminin isoforms can support the undifferentiated growth of human embryonic stem cells. Biochem Biophys Res Commun.

[16] Amit M, Shariki C, Margulets V, Itskovitz-Eldor J (2004). Feeder layer- and serum-free culture of human embryonic stem cells. Biol Reprod.

[17] James D, Levine AJ, Besser D, Hemmati-Brivanlou A (2005). TGFbeta/activin/nodal signaling is necessary for the maintenance of pluripotency in human embryonic stem cells. Development.

[18] Rodin S, Domogatskaya A, Strom S, Hansson EM, Chien KR, Inzunza J (2010). Long-term self-renewal of human pluripotent stem cells on human recombinant laminin-511. Nat Biotechnol.

[19] Hynes RO (2002). Integrins: bidirectional, allosteric signaling machines. Cell.

[20] Campbell ID, Humphries MJ. Integrin structure, activation, and interactions. Cold Spring Harb Perspect Biol. 2011;3(3). 10.1101/cshperspect.a004994 PMC3039929 21421922

[21] Campos L, Leone D, Revas J (2004). Beta1 integrins activate a MAPK signaling in neural stem cells that contributes to their maintenance. Development.

[22] Sutherland AE, Calarco PG, Damsky CH (1993). Developmental regulation of integrin expression at the time of implantation in the mouse embryo. Development.

[23] Stephens LE, Sutherland AE, Klimanskaya IV, Andrieux A, Meneses J, Pedersen RA (1995). Deletion of beta 1 integrins in mice results in inner cell mass failure and peri-implantation lethality. Genes Dev.

[24] Fassler R, Meyer M (1995). Consequences of lack of beta 1 integrin gene expression in mice. Genes Dev.

[25] Hayashi Y, Furue MK, Okamoto T, Ohnuma K, Myoishi Y, Fukuhara Y (2007). Integrins regulate mouse embryonic stem cell self-renewal. Stem Cells.

[26] Lee ST, Yun JI, Jo YS, Mochizuki M, van der Vlies AJ, Kontos S (2010). Engineering integrin signaling for promoting embryonic stem cell self-renewal in a precisely defined niche. Biomaterials.

[27] Ruoslahti E (1991). Integrins. J Clin Invest.

[28] Burdon T, Stracey C, Chambers I, Nichols J, Smith A (1999). Suppression of SHP-2 and ERK signaling promotes self-renewal of mouse embryonic stem cells. Dev Biol.

[29] Domogatskaya A, Rodin S, Boutaud A, Tryggvason K (2008). Laminin-511 but not -332, -111, or -411 enables mouse embryonic stem cell self-renewal in vitro. Stem Cells.

[30] Meng Y, Eshghi S, Li YJ, Schmidt R, Schaffer DV, Healy KE (2010). Characterization of integrin engagement during defined human embryonic stem cell culture. FASEB J.

[31] Rowland TJ, Miller LM, Blaschke AJ, Doss EL, Bonham AJ, Hikita ST (2010). Roles of integrins in human induced pluripotent stem cell growth on Matrigel and vitronectin. Stem Cells Dev.

[32] Horton ER, Humphries JD, James J, Jones MC, Askari JA, Humphries MJ (2016). The integrin adhesome network at a glance. J Cell Sci.

[33] Berrier AL, Yamada KM (2007). Cell-matrix adhesion. J Cell Physiol.

[34] Moser M, Legate KR, Zent R, Fassler R (2009). The tail of integrins, talin, and kindlins. Science.

[35] Harburger DS, Calderwood DA (2009). Integrin signaling at a glance. J Cell Sci.

[36] Frame MC, Patel H, Serrels B, Lietha D, Eck MJ (2010). The FERM domain: organizing the structure and function of FAK. Nat Rev Mol Cell Biol.

[37] Hall JE, Fu W, Schaller MD (2011). Focal adhesion kinase: exploring Fak structure to gain insight into function. Int Rev Cell Mol Biol.

[38] Calalb MB, Polte TR, Hanks SK (1995). Tyrosine phosphorylation of focal adhesion kinase at sites in the catalytic domain regulates kinase activity: a role for Src family kinases. Mol Cell Biol.

[39] Ceccarelli DF, Song HK, Poy F, Schaller MD, Eck MJ (2006). Crystal structure of the FERM domain of focal adhesion kinase. J Biol Chem.

[40] Giancotti FG, Ruoslahti E (1999). Integrin signaling. Science.

[41] Thomas JW, Ellis B, Boerner RJ, Knight WB, White GC, Schaller MD (1998). SH2- and SH3-mediated interactions between focal adhesion kinase and Src. J Biol Chem.

[42] Guan JL (2010). Integrin signaling through FAK in the regulation of mammary stem cells and breast cancer. IUBMB Life.

[43] Chen HC, Appeddu PA, Isoda H, Guan JL (1996). Phosphorylation of tyrosine 397 in focal adhesion kinase is required for binding phosphatidylinositol 3-kinase. J Biol Chem.

[44] Schlaepfer DD, Broome MA, Hunter T (1997). Fibronectin-stimulated signaling from a focal adhesion kinase-c-Src complex: involvement of the Grb2, p130cas, and Nck adaptor proteins. Mol Cell Biol.

[45] Streuli CH, Akhtar N (2009). Signal co-operation between integrins and other receptor systems. Biochem J.

[46] Huveneers S, Danen EH (2009). Adhesion signaling—crosstalk between integrins, Src and Rho. J Cell Sci.

[47] Burridge K, Guilluy C (2016). Focal adhesions, stress fibers and mechanical tension. Exp Cell Res.

[48] Strutchbury B, Atherton P, Tsang R, Wang D-Y, Ballestrem C (2017). Distinct focal adhesion protein modules control different aspects of mechanotransduction. J Cell Sci.

[49] Frisch SM, Francis H (1994). Disruption of epithelial cell-matrix interactions induces apoptosis. J Cell Biol.

[50] Gilmore AP, Metcalfe AD, Romer LH, Streuli CH (2000). Integrin-mediated survival signals regulate the apoptotic function of Bax through its conformation and subcellular localization. J Cell Biol.

[51] Meredith JE, Fazeli B, Schwartz MA (1993). The extracellular matrix as a cell survival factor. Mol Biol Cell.

[52] Frisch SM, Vuori K, Ruoslahti E, Chan-Hui PY (1996). Control of adhesion-dependent cell survival by focal adhesion kinase. J Cell Biol.

[53] Hungerford JE, Compton MT, Matter ML, Hoffstrom BG, Otey CA (1996). Inhibition of pp125FAK in cultured fibroblasts results in apoptosis. J Cell Biol.

[54] Cantley LC (2002). The phosphoinositide 3-kinase pathway. Science.

[55] Yu JS, Cui W (2016). Proliferation, survival and metabolism: the role of PI3K/AKT/mTOR signaling in pluripotency and cell fate determination. Development.

[56] Paling NR, Wheadon H, Bone HK, Welham MJ (2004). Regulation of embryonic stem cell self-renewal by phosphoinositide 3-kinase-dependent signaling. J Biol Chem.

[57] Wu CC, Wu HJ, Wang CH, Lin CH, Hsu SC, Chen YR (2015). Akt suppresses DLK for maintaining self-renewal of mouse embryonic stem cells. Cell Cycle.

[58] Armstrong L, Hughes O, Yung S, Hyslop L, Stewart R, Wappler I (2006). The role of PI3K/AKT, MAPK/ERK and NFkappabeta signaling in the maintenance of human embryonic stem cell pluripotency and viability highlighted by transcriptional profiling and functional analysis. Hum Mol Genet.

[59] Singh AM, Reynolds D, Cliff T, Ohtsuka S, Mattheyses AL, Sun Y (2012). Signaling network crosstalk in human pluripotent cells: a Smad2/3-regulated switch that controls the balance between self-renewal and differentiation. Cell Stem Cell.

[60] Hossini AM, Quast AS, Plotz M, Grauel K, Exner T, Kuchler J (2016). PI3K/AKT signaling pathway is essential for survival of induced pluripotent stem cells. PLoS One.

[61] Niwa H, Ogawa K, Shimosato D, Adachi K (2009). A parallel circuit of LIF signaling pathways maintains pluripotency of mouse ES cells. Nature.

[62] Wray J, Kalkan T, Gomez-Lopez S, Eckardt D, Cook A, Kemler R (2011). Inhibition of glycogen synthase kinase-3 alleviates Tcf3 repression of the pluripotency network and increases embryonic stem cell resistance to differentiation. Nat Cell Biol.

[63] Xia H, Nho RS, Kahm J, Kleidon J, Henke CA (2004). Focal adhesion kinase is upstream of phosphatidylinositol 3-kinase/Akt in regulating fibroblast survival in response to contraction of type I collagen matrices via a beta 1 integrin viability signaling pathway. J Biol Chem.

[64] Persad S, Attwell S, Gray V, Mawji N, Deng JT, Leung D (2001). Regulation of protein kinase B/Akt-serine 473 phosphorylation by integrin-linked kinase: critical roles for kinase activity and amino acids arginine 211 and serine 343. J Biol Chem.

[65] Attwell S, Roskelley C, Dedhar S (2000). The integrin-linked kinase (ILK) suppresses anoikis. Oncogene.

[66] Qin J, Wu C (2012). ILK: a pseudokinase in the center stage of cell-matrix adhesion and signaling. Curr Opin Cell Biol.

[67] Ozes ON, Mayo LD, Gustin JA, Pfeffer SR, Pfeffer LM, Donner DB (1999). NF-kappaB activation by tumour necrosis factor requires the Akt serine-threonine kinase. Nature.

[68] Zhang H, Saeed B, Ng SC (1995). Combinatorial interaction of human bcl-2 related proteins: mapping of regions important for bcl-2/bcl-x-s interaction. Biochem Biophys Res Commun.

[69] Stupack DG, Cheresh DA (2002). Get a ligand, get a life: integrins, signaling and cell survival. J Cell Sci.

[70] Ilic D, Almeida EA, Schlaepfer DD, Dazin P, Aizawa S, Damsky CH (1998). Extracellular matrix survival signals transduced by focal adhesion kinase suppress p53-mediated apoptosis. J Cell Biol.

[71] Jones G, Machado J, Merlo A (2001). Loss of focal adhesion kinase (FAK) inhibits epidermal growth factor receptor-dependent migration and induces aggregation of nh(2)-terminal FAK in the nuclei of apoptotic glioblastoma cells. Cancer Res.

[72] Golubovskaya VM, Finch R, Cance WG (2005). Direct interaction of the N-terminal domain of focal adhesion kinase with the N-terminal transactivation domain of p53. J Biol Chem.

[73] Golubovskaya VM, Cance WG (2008). FAK and p53 protein interactions. Anti Cancer Agents Med Chem.

[74] Lim ST, Chen XL, Lim Y, Hanson DA, Vo TT, Howerton K (2008). Nuclear FAK promotes cell proliferation and survival through FERM-enhanced p53 degradation. Mol Cell.

[75] Toya SP, Wary KK, Mittal M, Li F, Toth PT, Park C (2015). Integrin alpha6beta1 expressed in ESCS instructs the differentiation to endothelial cells. Stem Cells.

[76] Uda Y, Poh YC, Chowdhury F, Wu DC, Tanaka TS, Sato M (2011). Force via integrins but not E-cadherin decreases Oct3/4 expression in embryonic stem cells. Biochem Biophys Res Commun.

[77] Park J, Ryu J, Han H (2011). Involvement of caveolin-1 in fibronectin-induced mouse embryonic stem cell proliferation: role of FAK, RhoA, PI3K/Akt, and ERK 1/2 pathways. J Cell Physiol.

[78] Miyazaki T, Futaki S, Suemori H, Taniguchi Y, Yamada M, Kawasaki M (2012). Laminin E8 fragments support efficient adhesion and expansion of dissociated human pluripotent stem cells. Nat Commun.

[79] Wrighton PJ, Klim JR, Hernandez BA, Koonce CH, Kamp TJ, Kiessling LL (2014). Signals from the surface modulate differentiation of human pluripotent stem cells through glycosaminoglycans and integrins. Proc Natl Acad Sci U S A.

[80] Vitillo L, Baxter M, Iskender B, Whiting P, Kimber SJ (2016). Integrin-associated focal adhesion kinase protects human embryonic stem cells from apoptosis, detachment, and differentiation. Stem Cell Reports.

[81] Villa-Diaz LG, Kim JK, Laperle A, Palecek SP, Krebsbach PH (2016). Inhibition of focal adhesion kinase signaling by integrin alpha6beta1 supports human pluripotent stem cell self-renewal. Stem Cells.

[82] Afrikanova I, Yebra M, Simpkinson M, Xu Y, Hayek A, Montgomery A (2011). Inhibitors of Src and focal adhesion kinase promote endocrine specification: impact on the derivation of beta-cells from human pluripotent stem cells. J Biol Chem.

[83] Hayashi Y, Furue MK (2016). Biological effects of culture substrates on human pluripotent stem cells. Stem Cells Int.

[84] Ho B, Olson G, Figel S, Gelman I, Cance WG, Golubovskaya VM (2012). Nanog increases focal adhesion kinase (FAK) promoter activity and expression and directly binds to FAK protein to be phosphorylated. J Biol Chem.

